# Key protective factors that mitigate the impact of childhood trauma on poor mental health in adulthood: a scoping review protocol

**DOI:** 10.1136/bmjopen-2025-112097

**Published:** 2026-02-12

**Authors:** Anna Clarke, Nutmeg Hallett, Yvette Brown, Claire Cunnington, Maria R Dauvermann, Gerald Jordan, Renate Reniers, Julie Taylor

**Affiliations:** 1 School of Nursing and Midwifery, University of Birmingham, Birmingham, UK; 2 Institute for Mental Health, University of Birmingham, Birmingham, UK; 3 School of Sociological Studies, Politics and International Relations, University of Sheffield, Sheffield, UK; 4 School of Psychological Science, University of Bristol, Bristol, UK; 5 Birmingham Medical School, University of Birmingham, Birmingham, UK

**Keywords:** MENTAL HEALTH, Adverse events, Child

## Abstract

**Abstract:**

**Introduction:**

A significant proportion of adults in England and Wales report experiencing childhood trauma, which is often associated with poor health and negative social outcomes including a significant increase in the risk of poor mental health outcomes in adulthood. This proposed scoping review adopts a broad definition of childhood trauma and applies both a salutogenic framework and ecological systems theory to explore how protective factors at five ecological levels can support mental well-being. The review will also examine how protective factors vary across different population groups and contexts.

**Methods and analysis:**

The scoping review will follow the Joanna Briggs Institute (JBI) protocol for scoping reviews. The databases that will be searched are Embase, PubMed, Web of Science, PsycINFO, CINAHL and Medline. Studies will be included if they include protective factors and involve adults aged 18 and over who have experienced childhood trauma, whether self-identified, retrospectively self-reported or measured using a validated instrument. Studies will be excluded if they focus on participants under the age of 18.

All search results will be uploaded to Covidence, duplicates removed, and titles/abstracts screened by at least two reviewers based on inclusion criteria. Full texts of potentially relevant sources will be imported into EndNote 21. Reasons for exclusions will be documented and disagreements resolved through discussion or a third reviewer. The full process will be reported using a Preferred Reporting Items for Systematic Reviews and Meta-Analyses flow diagram. Data will be extracted by at least two reviewers using a tool developed by the team based on the JBI guidance. A best-fit framework analysis will be used, using a matrix developed by the researchers including the four salutogenic domains and the five levels of the ecological framework.

**Ethics and dissemination:**

Formal ethical approval is not necessary for this scoping review as it does not involve the collection of primary data. The outcomes of this study will be disseminated through peer-reviewed journal articles, conference/seminar presentations, and developed into resources for stakeholders and collaborators.

**Trial registration number:**

Open Science Framework (DOI 10.17605/OSF.IO/CJRUY).

**STRENGTHS AND LIMITATIONS OF THIS STUDY**

- The review will include an extensive literature search and rigorous screening process and will be conducted in accordance with guidelines for scoping reviews.
- The broad definition of childhood trauma ensures the applicability of the salutogenic approach across different population groups and contexts.
- A research advisory group consisting of people with lived experience of childhood trauma will be offering insight throughout the process of the review, including in the development of the search strategy which will increase quality and relevance.
- Papers included in the review will not be assessed for quality or risk of bias as per the guidelines for conducting scoping reviews.

## Introduction

### Childhood trauma

 Childhood trauma encompasses a wide range of adverse experiences occurring during childhood that can have enduring effects on mental health and well-being across the life course.^1^,^3^ Globally, childhood adversity is highly prevalent. Up to one billion children aged 2–17 years are estimated to experience physical, sexual or emotional violence or neglect each year, highlighting childhood trauma as a major public health concern across diverse countries and cultures.^4^ Approximately 20% of adults in England and Wales report having experienced at least one form of child abuse, including physical, emotional, sexual or neglect, by the age of 16.^5^

A substantial body of evidence demonstrates that exposure to childhood trauma is associated with an increased risk of poor mental health outcomes in adulthood, including depression, anxiety, post-traumatic stress disorder and psychosis.^1 6^ Childhood trauma has also been linked with greater symptom severity across a range of mental health conditions, including psychotic disorders, bipolar disorder and obsessive-compulsive disorder, as well as poorer cognitive and emotional functioning.^7^,^9^ Evidence consistently suggests that risk increases with the accumulation and severity of adverse experiences, underscoring the long-term impact of early life trauma on adult mental health.

One widely used approach to quantifying exposure to childhood adversity is the adverse childhood experience (ACE) checklist, originally developed to explore the relationship between childhood emotional, physical and sexual abuse and household dysfunction with adult health outcomes.^10^ Using this measure, nearly half of all adults in England and Wales have reported experiencing at least one ACE and approximately one in 10 report exposure to four or more, which is associated with substantially elevated risk of mental illness, health harming behaviours and non-communicable diseases.^1 11 12^

There is, however, ongoing contestation about whether the ACE framework adequately captures the breadth and complexity of childhood adversity. Critics have highlighted that ACE scores often fail to account for the severity, duration and subjective impact of experiences, such as equating parental mental illness with repeated sexual abuse.^13^ As such, ACEs are treated in this review as one measurement approach rather than a comprehensive definition of childhood trauma.

Accordingly, this scoping review adopts a broad conceptualisation of childhood trauma, drawing on the Office for Health Improvement and Disparities definition, which conceptualises trauma as resulting from ‘an event, series of events, or set of circumstances that is experienced by an individual as physically or emotionally harmful or life threatening’.^14^ This includes, but is not limited to, child abuse, maltreatment, neglect, parental loss, bullying, natural disasters, experiences of war and medical trauma. By adopting this definition, the review recognises that traumatic experiences extend beyond those captured in standardised checklists and vary widely across individuals, populations and contexts.

### Salutogenesis

The traditional pathogenic approach to childhood trauma focuses on the negative impact and consequences. While this approach is important and valuable, the pathogenic approach often fails to consider the resilience and coping mechanisms that survivors have developed following the experience of childhood trauma.

The salutogenic approach, originally conceptualised by Antonovsky, focuses on the factors and resources that enable individuals to manage stressors and move towards better health and well-being, viewing health and disease as a continuum.^15^ A key aspect of salutogenesis is the ‘sense of coherence’, which comprises three components: comprehensibility, the extent to which the world is perceived as ordered and problems are understood; manageability, the belief that sufficient coping resources are available; and meaningfulness, the extent to which problems and demands are seen as worthy of engagement and commitment.^15^

Antonovsky described a set of resources that contribute to the development of the individual’s sense of coherence as generalised resistance resources (GRRs). These include physical, biochemical, cognitive, emotional, valuative-attitudinal, interpersonal-relational, artefactual-material and macrosociocultural characteristics.^16^ These GRRs can be categorised into four main domains: the biological (physical and biochemical), psychological (cognitive and emotional), interpersonal (valuative-attitudinal and interpersonal-relational) and sociocultural (artefactual-material and macrosociocultural).^17^

Although salutogenic research has been applied across diverse contexts, its application to childhood trauma survivors remains limited despite existing findings highlighting its potential to enhance wellbeing.^18^ By focusing on a salutogenic framework, this scoping review aims to identify the protective factors that enable individuals exposed to childhood trauma to move towards health on the health/disease continuum.

### Protective factors

In addition to the salutogenic perspective, this review draws on Bronfenbrenner’s ecological systems theory (EST), which conceptualises health and well-being as shaped by dynamic interactions across multiple levels of influence.^19^ These include the microsystem (eg, family, peers and school), mesosystem (connections between immediate environments), exosystem (indirect settings that affect the individual, such as parental workplaces or healthcare access), macrosystem (cultural values, societal norms, policies) and chronosystem (changes over time, including life transitions and historical context). Drawing on this framework alongside the four salutogenic domains (biological, psychological, interpersonal and sociocultural) provides a comprehensive lens to investigate how protective factors operate across systems to support mental well-being and promote health from childhood into adulthood.

For the purpose of this review, protective factors are defined as individual, relational, community or societal attributes that increase resilience and positively reduce the impact of adversity.^14^ Such protective factors are considered through the dual lens of salutogenesis and EST. Within the salutogenic framework, protective factors are viewed as key resources within biological, psychological, interpersonal and sociocultural domains that support the development of a strong sense of coherence, enabling individuals to perceive their lives as comprehensible, manageable and meaningful. In parallel, Bronfenbrenner’s EST offers a multilayered view of how protective factors operate across nested systems, from individual traits and close relationships (microsystem) to institutional supports and cultural values (macrosystem). By integrating these frameworks, this review aims to investigate how protective factors interact across salutogenic domains and ecological systems to support the movement towards better mental health and well-being in survivors of childhood trauma.

### Variations across populations and contexts

The frequency and severity of childhood trauma vary significantly across individuals and different population groups. Socioeconomic status, ethnicity, cultural background, geographic location and exposure to conflict or displacement can all influence the likelihood and nature of traumatic experiences during childhood.^11 12^ Recognising these differences underlines the importance of understanding how protective factors vary across individuals, populations and contexts.

### Rationale

While recent literature reviews have examined the relationships between the experience of childhood trauma, protective factors and adult mental health outcomes,^20^,^22^ there remains a gap in knowledge of whether and how a salutogenic perspective may increase insight into the complex links between protective factors after the experience of childhood trauma and subsequent mental health outcomes across various individuals, populations and contexts.

The aim of this scoping review is to identify key protective factors that enable people to move towards better mental health and well-being after experiencing childhood trauma; and determine the extent to which these protective factors vary across different populations and contexts. This aim is based on the extensive research that has been conducted showing that experiencing childhood trauma significantly increases the risk of poor mental health outcomes in adulthood.^1^ Understanding the role of protective factors is essential as not all people who have experienced childhood trauma go on to develop mental illness. Even among individuals who do go on to develop mental illness, protective factors can substantially mitigate the impact of trauma and associated outcomes of illness, and in some cases, early adversity may foster enhanced resilience in later life.

## Review questions

1. What are the key protective factors that enable people to improve, maintain or restore their mental health after experiencing childhood trauma within a salutogenic and ecological framework?
2. To what extent do protective factors vary across different populations and contexts in their effectiveness of enabling people to improve, maintain or restore their mental health after experiencing childhood trauma within a salutogenic and ecological framework?

## Methods

The proposed scoping review will be conducted in accordance with the Joanna Briggs Institute (JBI) methodology for scoping reviews.^23^ The protocol is registered with Open Science Framework (DOI 10.17605/OSF.IO/CJRUY).

### Search strategy

The search strategy will aim to locate both published and unpublished studies. Search terms will include childhood trauma, early life adversity, abuse, neglect and ACEs, recognising that ACE terminology is widely used in the literature to index exposure despite conceptual limitations. A three-step search strategy will be used in this review. First, an initial limited search of Embase (Ovid) and PubMed was undertaken to identify articles on the topic. The key words contained in the titles and abstracts of relevant articles, and the index terms used to describe the articles were used to develop a full search strategy. This will be reviewed by a Research Advisory Group of people with lived experience and expert knowledge. The search strategy, including all identified keywords and index terms, will be adapted for each included database. The reference list of all included sources of evidence will be screened for additional studies. The search terms used for this review and example search are detailed in online supplemental appendix I.

To ensure a comprehensive synthesis of the available evidence, the selection of databases for the search prioritises breadth and coverage of relevant disciplines to capture a wide spectrum of peer-reviewed studies. The databases to be searched are: Embase (Ovid), PubMed, Web of Science, PsychInfo, CINAHL (Ovid) and Google Scholar. The abstracts of publications in languages other than English will also be screened, either by the research team if in languages fluent to them (French, Italian, Dutch or German) or by using artificial intelligence translation technology such as Google Translate.

### Eligibility criteria

Eligibility criteria have been developed using the population, concept, context framework to ensure a structured and transparent approach to study selection.^23^ The criteria were informed by preliminary mapping of the literature, which revealed substantial variation in how childhood trauma, protective factors and mental health outcomes are conceptualised and measured. To maintain a coherent and focused scope, only studies that explicitly examine the intersection of these three elements (childhood trauma, protective factors and mental health and well-being in adulthood) will be included.

### Population

Studies will be included if they involve adults aged 18 and over who have experienced childhood trauma, whether self-identified, retrospectively self-reported or measured using a validated instrument. Childhood trauma is conceptualised as the primary construct of interest in this review, as defined in the introduction. Measures based on ACEs are included as one approach to identifying exposure but are not treated as a comprehensive definition of childhood trauma.

Studies focusing exclusively on prenatal exposures, adversity occurring solely in adulthood or purely structural or socioeconomic disadvantage without a directly experienced traumatic component will be excluded. Studies focusing on children or adolescents under 18 years of age will also be excluded. A cross-sectional analysis will be undertaken if there are sufficient studies that include the emerging adulthood age range of 18–25 years old as brain maturation and development continues and is correlated with cognitive, emotional and social abilities in this population.^24^

### Concept

The core concept of interest is research examining the relationship between childhood trauma, protective factors and mental health outcomes in adulthood. Protective factors may operate by promoting positive mental health and well-being or by mitigating against the severity or impact of mental health symptoms. Within this review, reductions in symptom burden or distress are interpreted as movement towards health on a mental health continuum, consistent with a salutogenic perspective that emphasises manageability and functional adaptation rather than the binary presence or absence of disorder.^25^ Mental health outcomes may include, but are not limited to, psychological well-being, resilience, distress, emotional functioning or psychiatric diagnoses. Studies focusing solely on physical health outcomes, non-human populations or no reference to protective factors will be excluded.

### Context

This review will consider studies conducted in any geographical or sociocultural context, without restriction. Primary research of any design (eg, qualitative, quantitative, mixed methods) will be included as well as review articles if they contain new information. The reference lists of any potentially relevant reviews identified during screening will be searched to identify additional primary studies for inclusion. Study protocols and conference abstracts will be excluded.

### Study/source of evidence selection

Following the search, all results will be uploaded to Covidence Systematic Review Software, followed by automatic de-duplication. Following a pilot test, titles and abstracts will then be screened by two or more independent reviewers for assessment against the inclusion criteria for the review. Potentially relevant sources will be retrieved in full.

Two or more independent reviewers will assess the full texts of the selected citations in detail against the inclusion criteria. Excluded sources and reasons for exclusion will be documented. Reviewer disagreements will be resolved through discussion or with an additional reviewer. The search and study selection process will be fully reported in the final review and detailed in a Preferred Reporting Items for Systematic Reviews and Meta-Analyses flow diagram.^2^

### Data extraction

Data will be extracted from papers by two or more independent reviewers using a data extraction tool developed by the reviewers. Extracted data relating to childhood trauma exposure will record how trauma was operationalised, including whether ACE-based measures or alternative trauma definitions were used. The data extracted will include specific details about the participants, concept, context, study methods and key findings relevant to the review questions.

A draft data extraction form has been developed by the review team, adapted from the JBI methodology guidance for scoping reviews^3^ (see online supplemental appendix II). The draft tool will be piloted independently by two reviewers on a small sample of included studies to ensure clarity, consistency and relevance to the review questions. Following the pilot phase, one reviewer will complete data extraction for all included studies, and a second reviewer will independently extract data from a subset to verify accuracy and consistency.

The data extraction tool may be modified and revised as necessary during the data extraction process. Any modifications will be documented and reported in the final scoping review. Any disagreements or uncertainties during the data extraction process will be resolved through discussion among the reviewers or, if required, by consultation with a third reviewer.

### Data analysis and presentation

Best-fit framework analysis will be conducted,^26^ using a matrix based on the four salutogenic domains: biological, psychological, interpersonal and sociocultural, alongside Bronfenbrenner’s ECT, which conceptualises influence across five nested levels: microsystem, mesosystem, exosystem, macrosystem and chronosystem. This approach will allow protective factors to be mapped across domains and ecological levels regardless of whether exposure was identified using ACE measures or alternative trauma frameworks or definitions. An iterative approach will be taken. Data that does not align with the framework will be analysed thematically and the framework will be refined as needed to ensure it captures emerging patterns in the data.

### Patient and public involvement

This scoping review is part of a wider project involving an RAG made up of individuals who self-identify as having experienced childhood trauma. The advisory group will be involved throughout the process of the scoping review, offering insight to increase quality and relevance.

### Ethics and dissemination

Formal ethical approval is not necessary for this scoping review as it does not involve the collection of primary data. The outcomes of this study will be disseminated through peer-reviewed journal articles, conference/seminar presentations, and developed into resources for stakeholders and collaborators.

## Conclusion

The findings of the scoping review will be used to inform subsequent phases of a mixed-methods research project. By mapping the existing literature and identifying key protective factors, the review will help to identify current knowledge gaps and direct future lines of research. The involvement of individuals with lived experience throughout the process increases the potential for meaningful impact and for the use of findings to inform policies and guidance for supporting affected populations.

## Supplementary material

10.1136/bmjopen-2025-112097 online supplemental file 1
