# Supplementary material for: Key protective factors that mitigate the impact of childhood trauma on poor mental health in adulthood: a scoping review protocol
Source: BMJ Open. 2026 Feb 12;16(2):e112097. doi: 10.1136/bmjopen-2025-112097 (PMC12911740; doi:10.1136/bmjopen-2025-112097)
Supplement: online supplemental file 1 [file bmjopen-16-2-s001.docx]

# Appendices

### Appendix I: Search strategy

Example search strategy for Embase (via Ovid), conducted on 16/07/2025:

1. exp childhood adversity/ or exp early life stress/ or "ACEs".mp. or "traumatic childhood events".mp. or "abused children".mp. or "early traumatic events".mp. or "early childhood adversity".mp.
2. exp mental health/ or exp psychological well-being/ or exp emotional well-being/
3. exp "ethnic or racial aspects"/ or exp "gender and sex"/ or (exp economic status/ or exp educational status/ or exp income group/ or exp socioeconomic vulnerability/) or exp "groups by age and sex"/ or exp social background/
4. (prevention or protection or impact or reduc* or mitigat* or protective role).mp.
5. (protective factors or resilience or coping strategies or health-promoting factors or protective influences).mp. or exp coping/ or exp sense of coherence/ or exp "care and caring"/ or exp psychological adjustment/ or exp mindfulness/ or exp self concept/ or exp psychological resilience/ or exp health promotion/
6. 1 AND 2 AND 3 AND 4 AND 5

### Appendix II: Data extraction instrument

| Author(s) |  |
| --- | --- |
| Year of publication |  |
| Origin/country of origin |  |
| Study aim |  |
| Population and sample size |  |
| Methodology |  |
| Protective factors |  |
| Definition of childhood trauma used (e.g. ACE measure) |  |
| Outcome measures (if applicable) |  |
| Key findings related to the review question |  |
