# Supplementary material for: Key protective factors that mitigate the impact of childhood trauma on poor mental health in adulthood: a scoping review protocol
Source: BMJ Open. 2026 Feb 12;16(2):e112097. doi: 10.1136/bmjopen-2025-112097 (PMC12911740; doi:10.1136/bmjopen-2025-112097)
Supplement: Supplementary file 2 [file bmjopen-2025-112097.reviewer_comments.pdf]

PEER REVIEW HISTORY

BMJ Open publishes all reviews undertaken for accepted manuscripts. Reviewers are asked to complete a checklist review form and are provided with free text boxes to elaborate on their assessment. These free text comments are reproduced below.

ARTICLE DETAILS

Title (Provisional)

Key Protective Factors that Mitigate the Impact of Childhood Trauma on Poor Mental Health in Adulthood: A Scoping Review Protocol

Authors

Clarke, Anna; Hallett, Nutmeg; Brown, Yvette; Cunnington, Claire; Dauvermann, Maria R; Jordan, Gerald; Reniers, Renate; Taylor, Julie

---

VERSION 1 - REVIEW

**Reviewer**	**1**
**Name**	**Pollard, Rebecca**
**Affiliation**	**King's College London Institute of Psychiatry Psychology and Neuroscience, Department of Psychological Medicine**
**Date**	**27-Nov-2025**
**COI**	**None**

The manuscript aims to present a protocol for a scoping review examining protective factors that mitigate the impact of childhood trauma on later mental health outcomes, using a salutogenic and ecological framework. While the topic is important and the manuscript has several strengths - particularly its novel framing and strong methodological structure - significant clarifications are needed regarding key concepts, scope, and alignment of objectives.

Major Comments

1. Ambiguity in Definition and Scope of “Adverse Childhood Experiences (ACEs)”

The manuscript refers to “ACEs” repeatedly but does not specify which ACE framework or checklist is being used. It appears that the authors may be referencing the original Felitti et al. (1998) ACE scale, but this is not cited.

Later, the manuscript expands the list of eligible adverse experiences and includes constructs (e.g., child maltreatment, neglect, parental loss, bullying) that are part of the original ACE framework, creating conceptual overlap and confusion:

“This encompasses, but is not limited to, child abuse, ACEs, maltreatment, neglect, parental loss and bullying…”

This phrasing suggests ACEs are being treated as a separate category from maltreatment and neglect, which is inaccurate unless the authors are using an alternative ACE model.

Recommendation: Provide a precise definition of ACEs, reference the exact scale or framework being used, and clearly articulate the boundaries of the term “adverse experiences.” Clarify whether community- or area-level exposures (e.g., neighbourhood crime, deprivation) will be included, as this significantly affects scope. Also clarify whether other typically studied childhood trauma events such as institutionalisation, illness etc. are included in the scope

2. Conceptual Tension Between Salutogenic Framework and Focus on Poor Mental Health Outcomes

The review is positioned as adopting a salutogenic framework. However, the stated objective is:

“to identify… protective factors that mitigate the impact of childhood trauma on poor mental health in adulthood.”

This focus on poor mental health reflects a pathogenic rather than salutogenic orientation. Salutogenesis emphasises factors that promote positive adaptation and wellbeing, not merely the reduction of negative outcomes.

Similarly, throughout the protocol, outcomes are framed in terms of mental health disorders, symptoms, and risks. This needs clarification:

• Are the authors conceptualising “reduced negative outcomes” as consistent with a salutogenic approach?

• Will outcomes include positive indicators (e.g., wellbeing, resilience, competence)?

• How is salutogenesis operationalised in the context of trauma research?

Recommendation: Explicitly articulate how the salutogenic framework informs both the outcomes assessed and the interpretation of protective factors. Address whether studies focused on reducing negative symptoms (e.g., lower anxiety following trauma) are considered “salutogenic” within this review.

3. Lack of Explanation of the Four Salutogenic Domains and Their Role in the Review

Although the manuscript later references the four salutogenic domains (biological, psychological, interpersonal, sociocultural), these are not described in the introduction or linked conceptually to the review questions.

Recommendation: Introduce and briefly define these four domains early in the introduction to set the conceptual frame and provide the reader with clarity about why these domains matter and how they guide the review.

4. Review Questions Do Not Reflect the Salutogenic or Ecological Frameworks

The stated research questions do not mention either salutogenesis or ecological perspectives, even though these are presented as key conceptual underpinnings of the review. This weakens the coherence between the theoretical framing and the operationalisation of the review.

Recommendation: Revise the review questions to explicitly incorporate the salutogenic orientation and the ecological levels at which protective factors occur.

5. Understatement of Existing Literature on Protective Factors

The manuscript states:

"it is not well known what the deleterious or protective factors after the experience of childhood trauma are…"

However, there is an extensive literature on resilience and protective factors in relation to early life adversity (ELA), including factors at the individual, relational, and community levels, including numeous meta-analyses, systematic and umbrella reviews. The statement as written is misleading.

Recommendation: Revise to acknowledge the breadth of existing research, and specify more clearly what gap this scoping review fills (e.g., synthesising findings across several salutogenic domains, applying an ecological model.)

Strengths

• Novel framing: Focusing on salutogenesis and integrating an ecological framework is innovative and represents a shift from deficit-focused trauma research.

• Advisory group involvement: The use of a research advisory group enhances methodological rigour and relevance.

• Clear methodology: The methods are well-described, appropriate for a scoping review, and follow established frameworks such as PICO.

Minor Points & Suggestions

• Ensure consistent terminology when referring to childhood adversity, ACEs, trauma, and maltreatment.

• Provide examples of how salutogenic factors will be coded or categorised.

• Consider reorganising the introduction for clearer flow between adversity, protective factors, salutogenesis, and the rationale for a scoping review.

Conclusion

This manuscript addresses a meaningful and timely topic with strong methodological grounding and an innovative conceptual approach. To strengthen coherence and impact, it requires greater conceptual clarity, particularly around definitions of ACEs,

operationalisation of salutogenesis, and alignment between review aims and theoretical framework. With these refinements, the review could make a valuable contribution to the field.

VERSION 1 - AUTHOR RESPONSE

**Reviewer major comments**	**Response**
1. Ambiguity in Definition and Scope of "Adverse Childhood Experiences (ACEs)"<br>The manuscript refers to "ACEs" repeatedly but does not specify which ACE framework or checklist is being used. It appears that the authors may be referencing the original Felitti et al. (1998) ACE scale, but this is not cited.<br>Later, the manuscript expands the list of eligible adverse experiences and includes constructs (e.g., child maltreatment, neglect, parental loss, bullying) that are part of the original ACE framework, creating conceptual overlap and confusion:<br>"This encompasses, but is not limited to, child abuse, ACEs, maltreatment, neglect, parental loss and bullying..."<br>This phrasing suggests ACEs are being treated as a separate category from maltreatment and neglect, which is inaccurate unless the authors are using an alternative ACE model.<br>Recommendation: Provide a precise definition of ACEs, reference the exact scale or framework being used, and clearly articulate the boundaries of the term "adverse experiences." Clarify whether community- or area-level exposures (e.g., neighbourhood crime, deprivation) will be included, as this significantly affects scope. Also clarify whether other typically studied childhood trauma events such as institutionalisation, illness etc. are included in the scope	Explanation of ACEs provided and reference to the original Felliti et al., (1998) scale.<br><br>Phrasing changed in the introduction and methods to remove confusion around the inclusion of ACEs to introduce them as a widely used but heavily critiqued framework.<br><br>The scope of childhood trauma is being defined as: 'trauma results from an event, series of events, or set of circumstances that is experienced by an individual as physically or emotionally harmful or life threatening'. Further examples provided to highlight the breadth of inclusion medical trauma, war and natural disasters.
2) Conceptual Tension Between Salutogenic Framework and Focus on Poor Mental Health Outcomes<br>The review is positioned as adopting a salutogenic framework. However, the stated objective is:<br>"to identify... protective factors that mitigate the impact of childhood trauma on poor mental health in adulthood."<br>This focus on poor mental health reflects a pathogenic rather than salutogenic orientation. Salutogenesis emphasises factors that promote positive adaptation and wellbeing, not merely the reduction of negative outcomes.	The review questions and objective have been reworded to reflect the salutogenic framework.<br>Further explanation has been added to the concept section: *Protective factors may operate by promoting positive mental health and wellbeing or by mitigating against the severity or impact of mental health symptoms. Within this review, reductions in symptom burden or distress are interpreted as movement towards health on a mental health continuum,*

Similarly, throughout the protocol, outcomes are framed in terms of mental health disorders, symptoms, and risks. This needs clarification:<br>• Are the authors conceptualising “reduced negative outcomes” as consistent with a salutogenic approach?<br>• Will outcomes include positive indicators (e.g., wellbeing, resilience, competence)?<br>• How is salutogenesis operationalised in the context of trauma research?<br>Recommendation: Explicitly articulate how the salutogenic framework informs both the outcomes assessed and the interpretation of protective factors. Address whether studies focused on reducing negative symptoms (e.g., lower anxiety following trauma) are considered “salutogenic” within this review.	*consistent with a salutogenic perspective that emphasises manageability and functional adaptation rather than the binary presence or absence of disorder (Public Health Ontario, 2024).*
3. Lack of Explanation of the Four Salutogenic Domains and Their Role in the Review<br>Although the manuscript later references the four salutogenic domains (biological, psychological, interpersonal, sociocultural), these are not described in the introduction or linked conceptually to the review questions.<br>Recommendation: Introduce and briefly define these four domains early in the introduction to set the conceptual frame and provide the reader with clarity about why these domains matter and how they guide the review.	Explanation of the four salutogenic domains have been added to the introduction.
4. Review Questions Do Not Reflect the Salutogenic or Ecological Frameworks<br>The stated research questions do not mention either salutogenesis or ecological perspectives, even though these are presented as key conceptual underpinnings of the review. This weakens the coherence between the theoretical framing and the operationalisation of the review.<br>Recommendation: Revise the review questions to explicitly incorporate the salutogenic orientation and the ecological levels at which protective factors occur.	The review questions have been reworded to reflect the salutogenic framework and to mention the salutogenic and ecological framework explicitly.
5. Understatement of Existing Literature on Protective Factors<br>The manuscript states:<br>“it is not well known what the deleterious or protective factors after the experience of childhood trauma are…”<br>However, there is an extensive literature on resilience and protective factors in relation to early life adversity (ELA), including factors at the	The statement has been removed and further explanation added within the main text to emphasise the salutogenic approach.

individual, relational, and community levels, including numeous meta-analyses, systematic and umbrella reviews. The statement as written is misleading.<br>Recommendation: Revise to acknowledge the breadth of existing research and specify more clearly what gap this scoping review fills (e.g., synthesising findings across several salutogenic domains, applying an ecological model.)	

**Reviewer minor points and suggestions**	**Response**
Ensure consistent terminology when referring to childhood adversity, ACEs, trauma, and maltreatment.	Further clarification added about the definition of childhood trauma being used throughout the protocol.
Provide examples of how salutogenic factors will be coded or categorised.	This will be developed during the review.
Consider reorganising the introduction for clearer flow between adversity, protective factors, salutogenesis, and the rationale for a scoping review.	The introduction has been edited in response to the other comments and now has a clearer flow and rationale.

VERSION 2 - REVIEW

Reviewer 1

Name Pollard, Rebecca

Affiliation King's College London Institute of Psychiatry Psychology and Neuroscience, Department of Psychological Medicine

Date 05-Jan-2026

COI

The authors have made several constructive revisions to the manuscript in response to the reviewers' comments. The revised version demonstrates improved clarity regarding how the methodology and contextual framing of the review align with a salutogenic framework and an ecological systems perspective, and therefore how this review hopes to contribute novel insight to the existing literature on the topic. Revisions to the introduction including the addition of an overview of the salutogenic domains have improved conceptual clarity and the overall flow of the manuscript.

In addition, the improved phrasing of the definition of adversity to be used within the scoping review including how this will overlap with existing ACE scale is helpful and removes confusion.

Minor amendments

Please improve consistency in the terminology used to describe the theoretical framework. Within the manuscript, the authors refer to “ecological systems theory (SEM),” which introduces inconsistent nomenclature. A single, consistent term should be used throughout.

VERSION 2 - AUTHOR RESPONSE

Reviewer: 1
Miss Rebecca Pollard, King's College London Institute of Psychiatry Psychology and Neuroscience
Comments to the Author:
The authors have made several constructive revisions to the manuscript in response to the reviewers’ comments. The revised version demonstrates improved clarity regarding how the methodology and contextual framing of the review align with a salutogenic framework and an ecological systems perspective, and therefore how this review hopes to contribute novel insight to the existing literature on the topic. Revisions to the introduction including the addition of an overview of the salutogenic domains have improved conceptual clarity and the overall flow of the manuscript.
In addition, the improved phrasing of the definition of adversity to be used within the scoping review including how this will overlap with existing ACE scale is helpful and removes confusion.
Minor amendments
Please improve consistency in the terminology used to describe the theoretical framework. Within the manuscript, the authors refer to “ecological systems theory (SEM),” which introduces inconsistent nomenclature. A single, consistent term should be used throughout.

Dear Miss Rebecca Pollard,

Thank you for your review and comments. The manuscript has now been updated to improve consistency in the terminology used to describe the ecological systems theory framework. The term ecological systems theory (EST) has now been used instead of (SEM).
